# An affibody-adalimumab hybrid blocks combined IL-6 and TNF-triggered serum amyloid A secretion in vivo

**DOI:** 10.4161/mabs.36089

**Published:** 2014-11-01

**Authors:** Feifan Yu, Lindvi Gudmundsdotter, Anastassja Akal, Elin Gunneriusson, Fredrik Frejd, Per-Åke Nygren

**Affiliations:** 1 Division of Protein Technology; KTH Royal Institute of Technology; AlbaNova University Center; Stockholm, Sweden; 2 Affibody Medical AB; Gunnar Asplunds Allé 24; Solna, Sweden; 3 Department of Radiology, Oncology and Radiation Sciences; Uppsala University; Uppsala, Sweden

**Keywords:** AffiMab, Antibody, IL-6, TNF, adalimumab, affibody, affinity, fusion, inflammation

## Abstract

In inflammatory disease conditions, the regulation of the cytokine system is impaired, leading to tissue damages. Here, we used protein engineering to develop biologicals suitable for blocking a combination of inflammation driving cytokines by a single construct. From a set of interleukin (IL)-6-binding affibody molecules selected by phage display, five variants with a capability of blocking the interaction between complexes of soluble IL-6 receptor α (sIL-6Rα) and IL-6 and the co-receptor gp130 were identified. In cell assays designed to analyze any blocking capacity of the classical or the alternative (trans) signaling IL-6 pathways, one variant, Z*_IL-6_13_* with an affinity (K_D_) for IL-6 of ∼500 pM, showed the best performance. To construct fusion proteins (“AffiMabs”) with dual cytokine specificities, Z*_IL-6_13_* was fused to either the N- or C-terminus of both the heavy and light chains of the anti-tumor necrosis factor (TNF) monoclonal antibody adalimumab (Humira®). One AffiMab construct with Z*_IL-6_13_* positioned at the N-terminus of the heavy chain, denoted Z*_IL-6_13_*-HC_Ada_, was determined to be the most optimal, and it was subsequently evaluated in an acute Serum Amyloid A (SAA) model in mice. Administration of the AffiMab or adalimumab prior to challenge with a mix of IL-6 and TNF reduced the levels of serum SAA in a dose-dependent manner. Interestingly, the highest dose (70 mg/kg body weight) of adalimumab only resulted in a 50% reduction of SAA-levels, whereas the corresponding dose of the Z*_IL-6_13_*-HC_Ada_ AffiMab with combined IL-6/TNF specificity, resulted in SAA levels below the detection limit.

## Introduction

Inflammation is a cytokine-driven response by the innate immune system to destroy targets such as pathogens and damaged cells. In some disease conditions such as rheumatoid arthritis (RA) and Crohn's disease, the regulation of the inflammatory system is impaired, leading to tissue damages. Among the most studied inflammation inducers are the cytokines interleukin-6 (IL-6) and tumor necrosis factor (TNF). For TNF, numerous inhibitors are available for clinical use, such as the anti-TNF monoclonal antibodies adalimumab (Humira®) and infliximab (Remicade®) and the TNF receptor 2-Fc fusion protein etanercept (Enbrel®). For IL-6 driven conditions, the antibody tocilizumab (Actemra®), which binds to the IL-6 receptor α (IL-6Rα) rather than the cytokine itself, has been approved for clinical use. The choice between an IL-6 and TNF blocking anti-inflammatory therapeutic strategy in RA is not trivial. Whereas anti-TNF strategies have so far been considered standard, a recent comparative monotherapy Phase IV trial in RA patients showed that tocilizumab was more effective than adalimumab in reducing RA-related symptoms.^1^

**Figure 1.**
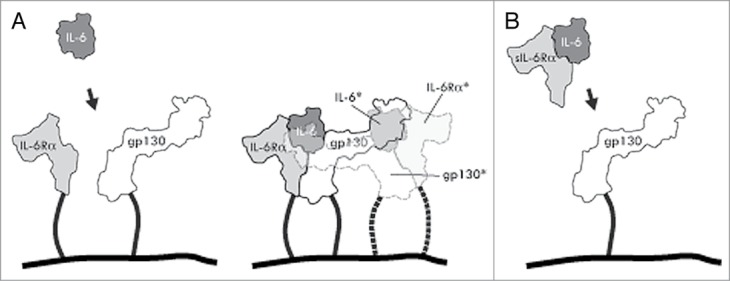
Schematic drawings of interleukin-6 signaling via classical and trans-signaling mechanisms. (**A**) Classical signaling. IL-6 binds to both a cell membrane bound IL-6 receptor α and gp130 to form a ternary complex (solid lines), that subsequently dimerizes with an adjacent ternary complex (semi-transparent; dashed lines and asterisked molecule names) necessary for signal transduction via the gp130 moieties. (**B**) Trans-signaling. A complex between sIL-6Rα and IL-6 binds to gp130 on cells not expressing the IL-6Rα.

IL-6 signaling and its regulation is complex, and involves a number of factors and mechanisms. In the so called classical IL-6 signaling mechanism, circulating IL-6 binds to a membrane-bound IL-6Rα, then a likewise membrane-anchored gp130 co-receptor is recruited. This results in formation of a ternary complex that subsequently homo-dimerizes with a second adjacent ternary complex, leading to signal transduction via the gp130 moieties (**Fig. 1A**).^2^ In circulation, IL-6 can also exist bound to soluble ectodomains of the IL-6 receptor α (sIL-6Rα), and these complexes cause the so-called trans-signaling mechanism driving IL-6 dependent activation of any cells expressing the co-receptor gp130 even though they may lack the IL-6R. The trans-signaling (or pro-inflammatory) pathway has been suggested to promote various disease conditions, and thus the most preferable to block. In contrast, the classical pathway is regarded to be responsible for important anti-inflammatory and regenerative processes.^3^ In addition to tocilizumab, other drug candidates are being developed to possibly address different IL-6 triggered pathways. These include antibodies CNTO136 (sirukumab)^4,5^ and MEDI5117,^6^ which both binding to the IL-6 cytokine itself, and a gp130-Fc fusion CR5/18 designed to selectively block the trans-signaling pathway.^7,8^

Considerable progress has recently been made in the construction of antibodies showing ability to bind to more than one antigen, for example through sophisticated engineering of the complementarity-determining regions (CDRs) to address two antigens in a single antibody combining site,^9,10^ via construction of heterodimeric antibodies using engineered Fc units^11-15^ or via genetic fusion of auxiliary recognition units to N- or C-terminus of light or heavy chains of full-length antibodies.^16,17^ One class of affinity proteins that have started to be investigated for the latter class of constructs are affibody binding proteins, which can be selected from combinatorial libraries of a 58-residues three-helix bundle domain to bind different target proteins with high affinity and selectivity.^18-21^ The efficient and independent folding of the small and non-cysteine containing affibody protein framework make them interesting to recruit as gene fusion partners to incorporate additional affinity units in larger proteins, including antibodies.^17^

In this work, we describe the generation and characterization of genetic fusions between IL-6 binding affibody binding proteins and the anti-TNF monoclonal antibody adalimumab for potential use in combined anti-IL-6 and anti-TNF blockade regimes. This includes the initial selections from a combinatorial library displayed on phage, functional screening of primary IL-6 binding variants by competition ELISA and cell assays, followed by in vivo experiments in a mouse model of both free affibody protein and affibody-adalimumab fusions.

## Results

### Selection and initial characterization of binders to human IL-6

**Figure 2.**
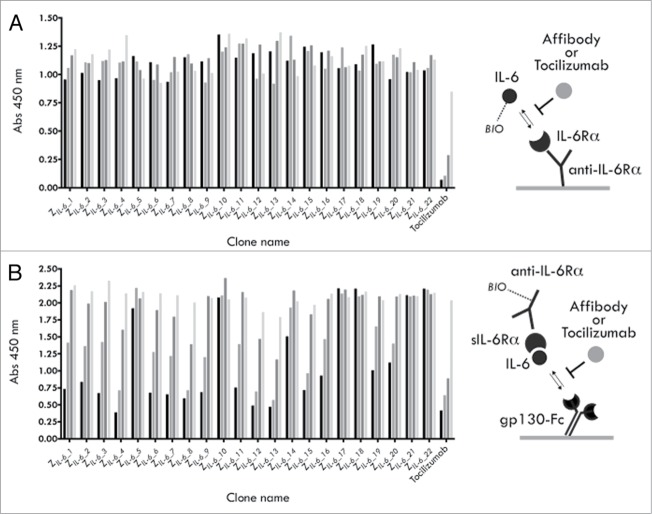
Competition ELISA experiments. (**A**) Results from an analysis of the ability of selected affibody variants, or the mAb tocilizumab, to compete with the interaction between IL-6 (biotinylated) and the IL-6Rα (**B**) Results from an analysis of the ability of selected affibody variants, or the mAb tocilizumab, to compete with the interaction between preformed IL-6 (biotinylated)/sIL-6Rα complexes and gp130 (gp130-Fc). For each construct, four concentrations were used: 500 nM (black bars), 50 nM, 5 nM and 0.5 nM (lightest gray bars).

A naïve affibody molecule library of 1.5 × 10^10^ complexity displayed on M13 filamentous phage (monovalent phagemid system) was used to select binders by biopanning to biotinylated hIL-6. After four selection rounds (see **Table S1**), a total of 372 candidate clones from all tracks were analyzed by ELISA, yielding 72 unique hIL-6 binding variants. Of this set, 22 representative variants were recloned and expressed as hexahistidine fusions in *E. coli*, purified by immobilized metal ion affinity chromatography (IMAC) and investigated further. Rather than performing rigorous affinity determinations to select prime candidates for further studies, a series of functional tests were instead performed to identify variants with the most interesting properties. In two separate competitive ELISA experiments, the capabilities of the selected IL-6 binders to block the interactions between either (1) IL-6 and the IL-6Rα or (2) gp130 and preformed hIL-6/shIL-6Rα complexes were investigated. Interestingly, none of the 22 tested variants showed any significant effect when tested for blocking of the IL-6/IL-6Rα interaction, whereas efficient blocking was seen for the anti-IL-6Rα antibody tocilizumab (**Fig. 2A**). On the contrary, 16 of the 22 variants instead showed a clear concentration-dependent blocking of the trans-signaling-resembling interaction between preformed IL-6/IL-6Rα complexes and gp130, where the five clones designated Z*_IL-6_4_*, Z*_IL-6_8_*, Z*_IL-6_12_*, Z*_IL-6_13_* and Z*_IL-6_15_* were the most efficient and in parity with tocilizumab (**Fig. 2B**). Taken together, these data suggest that the binding site for these 16 variants is located at the surface of the cytokine involved in the initial gp130 interaction (later forming the ternary sIL-6/IL-6/gp130 complex), rather than in the interaction between IL-6 and the IL-6Rα.

### Analysis of biological activity in cell assays

In two different cell assays, the five IL-6 binding affibody molecules Z*_IL-6_4_*, Z*_IL-6_8_*, Z*_IL-6_12_*, Z*_IL-6_13_* and Z*_IL-6_15_* identified in the ELISA experiment were investigated for their abilities to block IL-6 dependent signaling.

**Figure 3.**
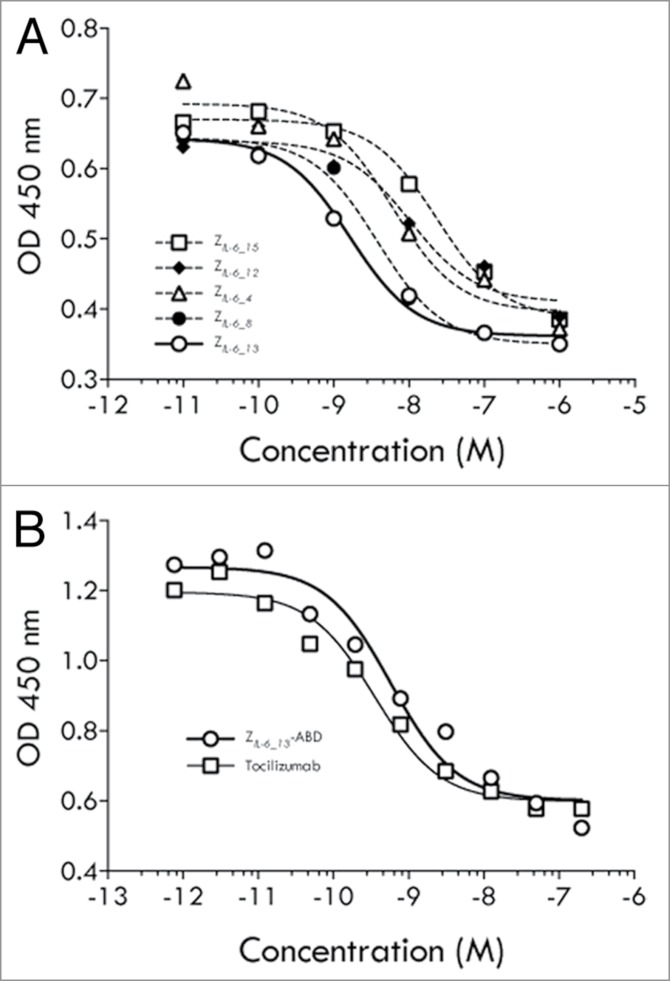
TF-1 cell assays (classical signaling). (**A**) Results from an analysis of the ability of affibody variants Z*_IL-6_4_*, Z*_IL-6_8_*, Z*_IL-6_12_*, Z*_IL-6_13_* and Z*_IL-6_15_*, expressed as His_6_-Z fusion proteins, to inhibit the growth of IL-6 dependent TF-1 cells. (**B**) Results from an analysis of the ability of affibody variant Z*_IL-6_13_*, expressed as a Z-ABD fusion protein, or the mAb tocilizumab, to inhibit the growth of IL-6 dependent TF-1 cells. The assay was performed in the presence of HSA.

TF-1 cells, responding to IL-6 by growth, were used to investigate if the IL-6 specific affibody molecules could block the classical signaling pathway. The assay showed that all five variants were capable of blocking IL-6 dependent growth of the TF-1 cells with approximate IC_50_ values ranging from mid- to low nanomolar (**Fig. 3A**). The variant showing the largest effect was the variant Z*_IL-6_13_* with an IC_50_ of ∼1.5 nM.

In a separate experiment utilizing the same assay set up, tocilizumab was compared with a half-life extended variant of Z*_IL-6_13_*. Here, the affibody molecule was produced as a fusion to a small (5 kDa) high affinity serum albumin binding domain (denoted ABD) designed to result in extended in vivo half-life via binding to circulating human serum albumin (HSA).^22,23^ This analysis revealed that the blocking effect for the half-life extended Z*_IL-6_13_* variant was retained with an approximate IC_50_ value of 1 nM (**Fig. 3B**). The high affinity anti-IL-6Rα antibody tocilizumab showed a higher potency in this assay, with an approximate IC_50_ value of 0.1 nM.

**Figure 4.**
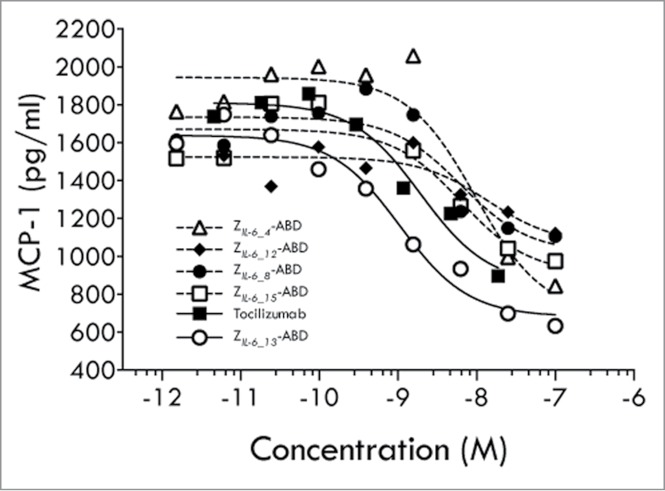
HUVEC cell assay (trans-signaling). Results from an analysis of the ability of affibody variants Z*_IL-6_4_*, Z*_IL-6_8_*, Z*_IL-6_12_*, Z*_IL-6_13_* and Z*_IL-6_15_*, expressed as Z-ABD fusion proteins, or the mAb tocilizumab, to block the IL-6 triggered secretion of MCP-1 from HUVEC cells.

To investigate if the trans-signaling pathway could be blocked in a cell-based system, gp130 positive and IL-6 receptor negative human umbilical vein endothelial cells (HUVECs) were used. Incubation of such cells with preformed IL-6/sIL-6Rα complexes results in IL-6 trans-signaling dependent secretion of monocyte chemoattractant protein-1 (MCP-1), thereby allowing analyses of trans-signaling blocking capabilities of IL-6 inhibitory molecules. Interestingly, all five investigated affibody variants were capable of inhibiting trans-signaling, albeit with different efficiencies (**Fig. 4**). Notably, in this assay the variant Z*_IL-6_13_* was again the most efficient variant and even showed to be more potent than tocilizumab, as judged from their observed approximate IC_50_ values of 1 and 5 nM, respectively.

### Design and in vitro characterization of affibody-mAb fusion proteins for combined IL-6 and TNF blocking

In inflammatory rheumatic diseases, competitive blocking of IL-6 induced effects has shown to be highly effective.^24^ Similarly, the high affinity anti-human TNF antibody adalimumab is clinically used for treatment of TNF induced inflammation indications, including rheumatoid arthritis (RA).^25^ To investigate if these two anti-inflammatory effects could be combined in one molecule, four different Z*_IL-6_13_*-adalimumab fusions were designed and produced. To this end, the anti-IL-6 Z*_IL-6_13_* affibody molecule moiety was genetically fused, via flexible (GGGGS)_3_ linkers, to either the N-terminus of the heavy (HC) or the light (LC) chain of adalimumab (Ada), resulting in the constructs Z*_IL-6_13_*-HC_Ada_ and Z*_IL-6_13_*-LC_Ada_, respectively, or to the C-terminus of the same chains, resulting in the constructs HC_Ada_-Z*_IL-6_13_* and LC_Ada_-Z*_IL-6_13_*, respectively (**Fig. S3A**). Analysis of the different AffiMab constructs by SDS-PAGE showed that they were of high purity and that the individual subunits showed expected size shifts in concordance with the Z*_IL-6_13_* affibody fusion site (**Fig. S3B**).

**Figure 5.**
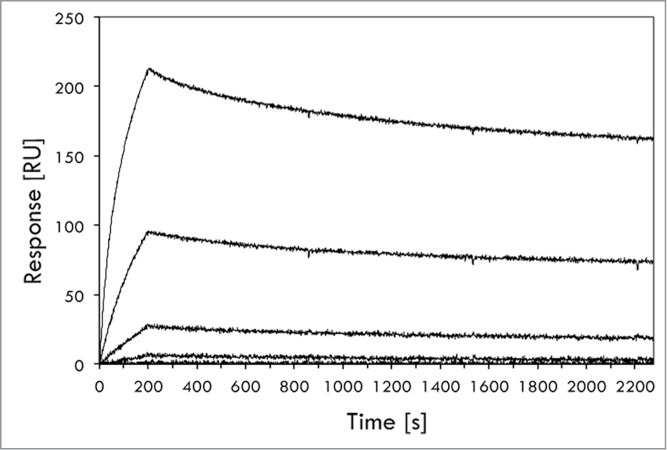
Biosensor-based affinity determination of the Z*_IL-6_13_* variant for IL-6. The affinity of affibody variant Z*_IL-6_13_* to IL-6 was determined in a ProteOn biosensor instrument (BioRad). The affibody variant, expressed as an His_6_-Z fusion, was immobilized onto a sensorchip via primary amine coupling chemistry followed by injections of different concentrations (0.08, 0.40, 2.0, 10 and 50 nM) of IL-6 and the responses recorded (see Materials and Methods section for details). The experiment was performed in triplicate, using separate sensor chip surfaces. A representative set of response traces (background buffer effect subtracted) are shown in the figure. The affinity (K_D_) was in this experiment determined to 4.97 **±** 0.3 × 10^−10^ M, i.e., ∼0.5 nM (500 pM).

To characterize the constructs further, affinity determinations to the two target proteins in questions, IL-6 and TNF, were performed using surface plasmon resonance technology for the original Z*_IL-6_13_* variant, the native adalimumab and two of the AffiMabs. The affinity (K_D_) of the “free” Z*_IL-6_13_* variant, expressed as a His_6_-tag fusion protein, for hIL-6 was determined to 500 ± 30 pM, which is relatively high taking into account that the selection was performed using a naïve library (**Fig. 5****; Table S4**). The affinity for TNF of the native adalimumab construct produced here was determined to 216 ± 1 pM, in parity with the approximate 100 pM value reported by the team that developed the antibody.^26^ For the Z*_IL-6_13_*-HC_Ada_ and the LC_Ada_-Z*_IL-6_13_* constructs containing the Z*_IL-6_13_* affibody moiety either close to the CDR regions or at the C-terminal end of the kappa light chain, respectively, the observed affinities for TNF were 170 ± 2 pM and 131 ± 3 pM. This indicates that the presence of the affibody in these constructs did not affect the affinity for TNF negatively. When analyzed for the affinity to IL-6, the affibody moiety in Z*_IL-6_13_*-HC_Ada_ and LC_Ada_-Z*_IL-6_13_* constructs showed to be only marginally affected, with observed dissociation constants of 720 ± 80 pM and 925 ± 100 pM, respectively (**Table S4**).

**Figure 6.**
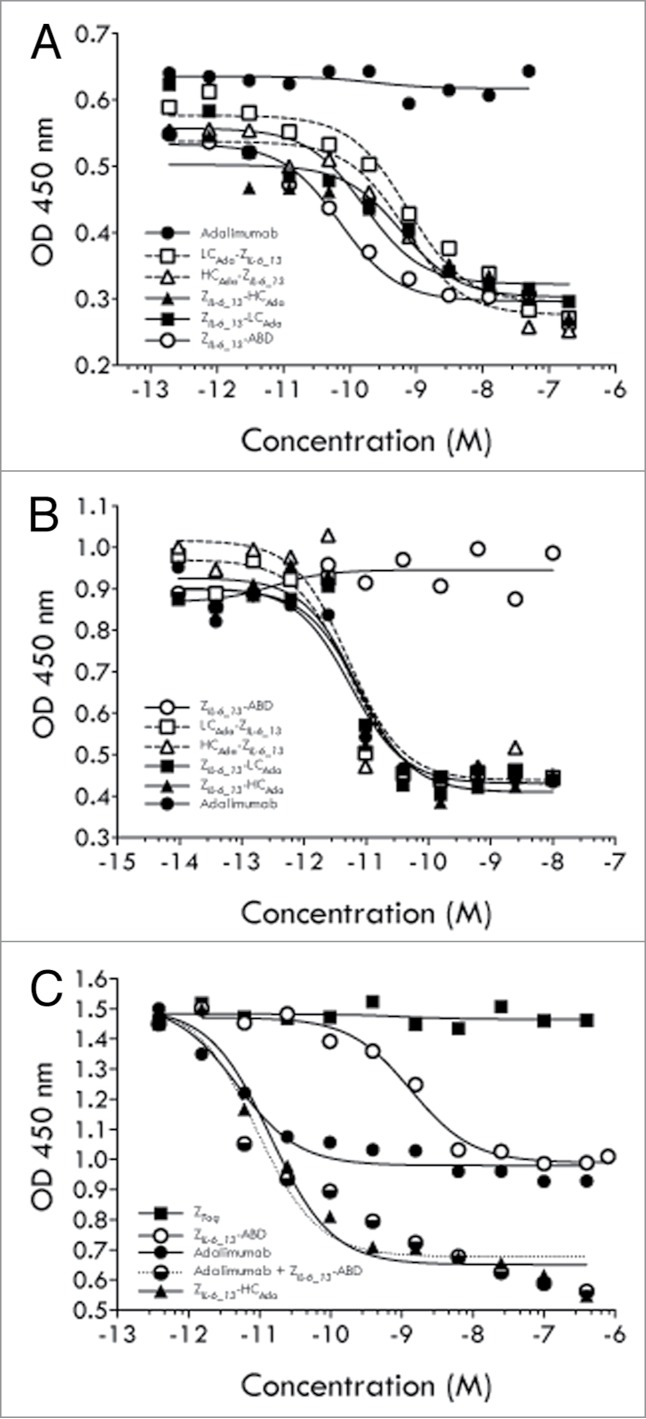
Characterization of AffiMabs in cell assays. (**A**) Results from an analysis of the ability of different proteins, as indicated, to inhibit the growth of IL-6 triggered TF-1 cells. (**B**) Results from an analysis of the ability of different proteins, as indicated, to inhibit the growth of TNF triggered TF-1 cells. (**C**) Results from an analysis of the ability of different proteins, as indicated, to inhibit the growth of TF-1 cells simultaneously triggered with IL-6 and TNF. See text for details.

The four different AffiMab constructs were evaluated for their capacity to block IL-6 or TNF dependent cells. As controls, both free adalimumab and free Z*_IL-6_13_*-ABD protein were included. All four AffiMab constructs, as well as the Z*_IL-6_13_*-ABD construct, showed growth inhibition capability in the IL-6 (99 pM) assay, with IC_50_ values in the range of 0.1 to 1 nM (**Fig. 6A**). As expected, adalimumab did not have any effect in the IL-6 blocking assay. In the experiment where TNF (23 pM) was used to stimulate the cells, all four AffiMabs, as well as adalimumab itself, showed effective blocking of the growth stimulating TNF signal, all with IC_50_ values around 10 pM (**Fig. 6B**). However, in a separate experiment performed at ∼3-fold higher TNF concentration (71.5 pM), the Z*_IL-6_13_*-HC_Ada_ AffiMab showed better blocking efficacy than the Z*_IL-6_13_*-LC_Ada_ construct (**Fig. S5**). As expected, the solely IL-6 binding Z*_IL-6_13_*-ABD fusion did not show any effect in TNF challenge assay.

In a subsequent set of experiments, TF-1 cells were simultaneously stimulated with both IL-6 (99 pM) and TNF (23 pM), and again the blocking effect of different constructs was investigated, including the Z*_IL-6_13_*-HC_Ada_ AffiMab construct, free adalimumab, Z*_IL-6_13_*-ABD protein and a mix of adalimumab and Z*_IL-6_13_*-ABD protein with a 1:2 (mAb:Z) ratio. A control affibody molecule construct, Z*_Taq_*-ABD that binds an irrelevant target (*Taq* DNA polymerase) was also included. The results showed that free adalimumab and Z*_IL-6_13_*-ABD protein both showed 50% growth inhibition compared with the maximum response, suggesting that approximately equal cell growth stimulating effects were coming from IL-6 and TNF, respectively (**Fig. 6C**). Interestingly, when adalimumab and Z*_IL-6_13_*-ABD protein was added together in a mix (1:2 mAb:Z ratio), full blocking of the cell growth could be observed, suggesting an additive blocking effect. A similarly strong effect was observed for the anti-IL-6 and anti-TNF AffiMab construct Z*_IL-6_13_*-HC_Ada_, showing that both its components were functional and independently could contribute to the cell growth inhibition as expressed in this combined format (**Fig. 6C**). No effect was seen for the control affibody molecule protein (**Fig. 6C**).

### In vivo study of the Z_IL-6_13_-HC_Ada_ AffiMab for combined IL-6 and TNF blocking

A mouse Serum Amyloid A (SAA) mouse model was utilized to study inflammation blocking effects in vivo. The acute phase protein SAA is secreted from liver cells and can be induced by the pro-inflammatory cytokines IL-1, IL-6, and TNF. Due to the sequence homology of the human and mouse cytokines, the human variants are able to act on their corresponding mouse receptors and induce a murine SAA response. The human TNF protein is, however, only able to interact with murine TNFRII (not murine TNFRI).

**Figure 7.**
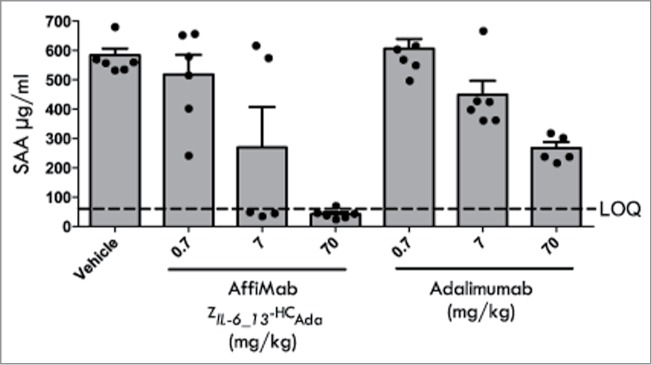
Analysis of the therapeutic effect of the AffiMab Z*_IL-6_13_*-HC_Ada_ hybrid in an acute SAA model. The AffiMab Z*_IL-6_13_*-HC_Ada_, corresponding to a hybrid construct between the anti-IL-6 affibody Z*_IL-6_13_* and the anti-TNF antibody adalimumab was analyzed in a mouse model for acute Serum amyloid A (SAA). For comparison, the effect of unmodified adalimumab was also tested in parallel. AffiMab or adalimumab doses of different concentrations were given nine hours before the animals (groups of six) were injected i.p. with a mixture of 2.5 μg/kg body weight each of TNF and IL-6 to trigger SAA production, which was analyzed 16 h post TNF/IL-6 injection. See text for details.

Results from an initial in vivo study performed with the half-life extended Z*_IL-6_13_* variant (Z*_IL-6_13_*-ABD) administered subcutaneously (s.c.) revealed a dose-dependent inhibition of the IL-6 induced SAA response (**Fig. S2**). Encouraged by this study, the AffiMab concept was investigated in the acute SAA model. Here, animals were challenged by a mixture of IL-6 and TNF, after having first been given varying doses i.p. of either the combined anti-IL-6 / anti-TNF AffiMab construct Z*_IL-6_13_*-HC_Ada_ or adalimumab. The results showed a complete inhibition of the cytokine induced SAA-response by the AffiMab construct, while the same dose of the TNF-blocking antibody alone only resulted in a 50% inhibition (**Fig. 7**). This suggests that the combination of the IL-6 signaling blocking Z*_IL-6_13_* affibody and the TNF-blocking adalimumab antibody in one molecule had generated a hybrid construct capable of dual action, resulting in a therapeutic relief to both the IL-6 and the TNF challenges.

## Discussion

The selection of affibody molecules against IL-6 resulted in a large number of IL-6 binding clones of which several, in competition ELISA experiments, were capable of interfering with the interaction between IL-6Rα/IL-6 complexes (“Site 2” on IL-6) and gp130, but none with the IL-6Rα/IL-6 interaction (“Site 1” on IL-6). It can be speculated that the observed bias toward interference with this interaction may reflect properties of the target protein and its different surfaces that may be more or less easy to address by in vitro affinity selection using the affibody molecule library. A structure of IL-6 in solution determined by nuclear magnetic resonance spectroscopy shows that the face of the cytokine facing the gp130 binding partner in the complex structure shows considerably less molecular motion than the opposite face recognized by the IL-6 receptor, which may have positively affected the selection of binders toward this face.^2,27^ Interestingly, a recently described, and also in vitro selected, nucleic acid aptamer to IL-6 has been shown to preferentially bind to the gp130 interacting surface characterized by this more rigid nature, although the aptamer structure also extends into the IL-6Rα/IL-6 interaction interface enough to affect that interaction.^28,29^ However, from a library of antibody-like binders called “minibodies,” binders to IL-6 have been selected that interfere with the IL-6Rα/IL-6 interaction, suggesting that the regions of IL-6 showing the increased mobility may indeed be addressed by in vitro selection.^30,31^ The IL-6 signal generating complex involves the formation of a hexameric structure that can be obstructed also by other means. In the complex formation, a third site on IL-6 is also involved (“Site 3”) and it has been shown to overlap with the epitope for the humanized anti-IL-6 monoclonal antibody olokizumab, initially discovered from screening of a massive number (ca. 10^9^) of supernatants of B-cells from immunized rats. Upon binding to IL-6, this antibody is able to induce a conformational change of the IL-6, further affecting the formation of a signaling competent complex.^32^

In cell assays, the Z*_IL-6_13_* affibody molecule was shown to be capable of blocking both the classical and trans-signaling pathways. As could potentially be expected from their respective binding profiles, the Z*_IL-6_13_* affibody capable of binding to preformed sIL-6Rα/IL-6 complexes was more effective in blocking trans-signaling than tocilizumab, which binds to IL-6Rα.

Four different Z*_IL-6_13_* affibody:adalimumab hybrids AffiMabs were constructed where the affibody moiety was positioned at different locations. In general, the relative positioning of the affibody molecule and the design of linkers used to connect affibody molecules and antibody subunits can be expected to influence the binding properties and performance of the resulting fusion construct. Depending on sterical factors, including the size of the respective antigens for the antibody and affibody molecule, the relative local importance of particular CDRs in the antibody or any biases in importance toward any of the two randomized helices of the affibody moiety will play a role. Also, each of the two antigens may either be present in solution or associated with a cell surface, or they may be engaged in homo- or hetero complexes, which affects their presentation and consequently the likelihood of both being engaged by a single AffiMab arm, a single AffiMab molecule or by two different AffiMab molecules, thereby affecting the observed effects. For example, it has earlier been described for fusions to the anti-HER2 antibody trastuzumab that the affinities of an angiopoietin-2 (Ang2)-specific disulfide constrained peptide and two different affibody molecules for their respective targets were negatively affected from being positioned C-terminally of the light chain.^17^ In this study, all four AffiMab constructs showed similar behavior in the TNF challenge cell assay (23 pM TNF), but the two AffiMabs corresponding to a C-terminal positioning of the affibody moiety at either the light or heavy chain showed somewhat higher IC_50_ values in the IL-6 challenge assay. In selecting an AffiMab construct for in vivo studies, we reasoned that an N-terminal positioning of the Z*_IL-6_13_* affibody moiety relative the heavy or light chain of adalimumab was preferable for sterical reasons because the first two α-helices of the affibody scaffold mediating the binding to IL-6 would be more accessible if positioned in front of another domain. This format also resembles the fusion orientation relative to other domains in which the affibody molecules are selected as presented on the phage particles. Here, the choice of the Z*_hIL-6_13_*-HC_Ada_ Affimab construct over the Z*_hIL-6_13_*-LC_Ada_ construct for the in vivo experiment was based on a somewhat better performance in an additional TNF challenge experiment using a higher cytokine concentration.

The TF-1 cell assay where the two cytokines had been mixed for a combined challenge showed that the Z*_IL-6_13_*-HC_Ada_ construct was capable of providing a cytokine-blocking effect in parity to a mix of its two free constituents, adalimumab and the Z*_IL-6_13_* affibody, here provided as an ABD fusion. In the subsequent animal study performed in mice, it was seen that this dual cytokine blocking effect also was effective in vivo, resulting in a therapeutic effect. In fact, two in vivo experiments were performed in mice, both utilizing a cytokine-triggered acute serum amyloid A secretion model. The first of the two studies (**Fig. S2**) was designed to primarily investigate if the Z*_IL-6_13_*-ABD construct would show any therapeutic effect at all in vivo relative to an irrelevant affibody control construct, and if so, if a dose dependent response could be seen. The second experiment was designed as a direct comparative study between the bi-specific Z*_IL-6_13_*-HCAda AffiMab and the parental TNF-specific adalimumab antibody concerning a double cytokine challenge. Here, to keep the number of animals involved to a minimum while still obtaining statistically valuable information it was prioritized to include more animals per group rather than including additional constructs such as the Z*_IL-6_13_*-ABD construct, for which positive in vivo efficacy data had already been obtained. In the second study, the doses of biologicals given in mg per kg body weight were higher, but we estimate that the molar ratios between these and the injected cytokines should still be comparable for the two studies, taking into account the combined effect from the more than 10-fold larger molecular weight of the full-length antibody format which on the other hand contains two Z*_IL-6_13_* affibody units per molecule, the injection of lower cytokine amounts in the second study, and use of either subcutaneous or intraperitoneal administration routes, associated with roughly estimated bioavailabilities of 35% and 80%, respectively.

The described study demonstrates the possibility to combine two anti-cytokine modalities in one antibody-based construct to provide a dual-action biological, and strengthens the notion that auxiliary binding units based on the affibody molecule class are suitable for this. Further optimizations of the Z*_IL-6_13_*-HC_Ada_ AffiMab construct, for example through affinity maturation of the IL-6 binding moiety or through linker length optimization, may provide variants of even higher potency.

## Materials and Methods

### Selection of affibody molecules from a naïve synthetic library

Human IL-6 (hIL-6, Peprotech), was biotinylated using No-Weigh Sulfo-NHS-LC-Biotin (Thermo Scientific) and dialyzed to remove unbound biotin. A combinatorial library of affibody molecules displayed on bacteriophage was subjected to four rounds of selection using biotinylated hIL-6 (bio-hIL-6) essentially according to Grönwall et al.^33^ The selection buffer consisted of phosphate buffered saline (10 mM phosphate, 137 mM NaCl, 2.68 mM KCl, pH 7.4, PBS) supplemented with 0.1% Tween 20 (PBST) and 3% BSA (Sigma), and PBST was used as wash buffer. Phage particles bound to biotinylated target were captured on streptavidin-coated paramagnetic beads before washing and phage particles were eluted at pH 2.2 (50 mM glycine-HCl). In general, for all tracks the target concentration was decreased and the number of washes was increased for each round of selection (see **Table S1**). The DNA sequences of the enriched clones were determined via PCR amplification of insert sequences and using an ABI PRISM® 3130xl Genetic Analyzer instrument (PE Applied Biosystems) according to the manufacturer's instructions.

### Soluble protein production from phagemid vectors and ELISA screen

Single colonies of *Escherichia coli (E. coli)* containing phagemids were cultivated in 96 deep-well plates with addition of 1 mM isopropyl-β-D-1-thiogalactopyranoside (IPTG) to induce production of soluble affibody molecules in fusion to a C-terminally positioned albumin binding domain (ABD). Bacteria containing the affibody-ABD fusions were subjected to repeated freeze-thawing and pelleted in a centrifuge to extract the proteins in the periplasmic fractions. Binding to hIL-6 was investigated in a standard enzyme-linked immunosorbent assay (ELISA). In brief, half area 96-well plates were coated with a polyclonal goat anti-ABD Ig (in-house produced) at 2 μg/ml. Soluble affibody-ABD variants were captured by incubation of periplasmic samples followed by addition of bio-hIL-6 at 200 ng/ml. Plates were developed by addition of horse-radish peroxidase-conjugated streptavidin diluted 1:30,000 (streptavidin-HRP, Thermo Scientific) and TMB (Thermo Scientific). Plates were measured at 450 nm using a microplate reader (Victor3, Perkin Elmer).

### Subcloning and production of affibody variants

A selection of 22 IL-6 binding affibody variants was subcloned to a vector with an N-terminally positioned hexahistidine tag under the control of a T7 promoter. These affibody variants were successfully purified from cell pellets by standard IMAC procedures under either native or denaturing conditions using Co^2+^-loaded Talon resin (Clontech). Six of the variants binders were also subcloned to a T7 promoter-containing vector as fusion to a C-terminally positioned engineered high affinity ABD variant.^22^ All variants were cultivated in *E. coli* BL21(DE3) at 37 °C in a multifermentor system (Greta, Belach Bioteknik), induced with IPTG and purified using affinity chromatography employing the ABD tag.

### Competitive ELISA assays

In a first assay, half area high binding EIA/RIA plates (Costar) were coated with anti-IL-6Rα capture antibody (RnD Systems) at a concentration of 2 μg/ml. Plates were incubated at +4 °C overnight, then washed twice in tap water. Plates were blocked for 1 h in PBS containing 0.5% casein (PBSC). IL-6Rα (RnD Systems) was added at a concentration of 250 ng/ml. Plates were incubated for 1.5 h at room temperature and then washed. In separate plates, serial dilutions of the 22 His_6_-Z proteins, as well as tocilizumab, were prepared from 615 nM to 0.615 nM with a dilution factor of 10 in PBSC buffer and a fixed concentration of bio-IL-6 of 0.615 nM (12.5 ng/ml) was added. The pre-mixed complex of the affibody molecules and bio-IL-6 were mixed for 15 min and then transferred to wells containing IL-6Rα. Plates were incubated for a further 1.5 h and then washed four times. A 1:8,000 dilution of streptavidin-HRP (Thermo Scientific) was added and the plates were incubated for 45 min. Plates were washed a final four times and TMB substrate (Thermo Scientific) was added for 15 min and the reaction was stopped with 2 M H_2_SO_4_. The absorbance was measured at 450 nm using a microplate reader. In a second assay, half area high binding enzyme/radio immunoassay (EIA/RIA) plates (Costar) were coated with a gp130-Fc fusion protein (RnD Systems) at a concentration of 3.5 μg/ml. Plates were incubated at +4 °C overnight and then washed twice in tap water. Plates were blocked for 1 h in PBSC. In separate plates serial dilutions of the His_6_-Z proteins, as well as tocilizumab, were titrated from 1 μM to 1 nM with a dilution factor of 10 in PBSC buffer with a fixed concentration of an IL-6/IL-6Rα solution (1 nM and 10 nM, respectively). The pre-mixed complex of the affibody molecules and IL-6/IL-6Rα was after 15 min incubation transferred to wells containing gp130-Fc. Plates were incubated for a further 1.5 h followed by washing four times. A biotinylated anti-IL-6Rα antibody (RnD Systems) at a concentration of 200 ng/ml was added and the plates were incubated for 1.5 h and then washed. A 1:8000 dilution of streptavidin-HRP was then added and the plates were incubated for 45 min. Plates were washed a final four times and TMB substrate was added. After 15 min the reaction was stopped with 2 M H_2_SO_4_ and the absorbance was measured at 450 nm in a microplate reader.

### In vitro neutralization assays

Cell assays employing TF-1 cells, were used to assess the blocking of the classical IL-6 signaling mechanism, or of TNF-stimulated growth. Cells were cultured in RPMI1640 with L-glut (Lonza) supplemented with 10% FCS (Gibco), Pen-Strep (Lonza) and 2 ng/ml recombinant human granulocyte macrophage colony-stimulating factor (rhGM-CSF, RnD Systems). Prior to analysis, cells were washed twice in RPMI1640 in the absence of rhGM-CSF. Cells were dispensed into 96-well flat-bottomed plates at a density of 4 × 10^4^/cells per well. In separate plates, serial dilutions of the potentially inhibitory compounds, i.e., affibody proteins, tocilizumab (Roche), AffiMab molecules or adalimumab, or mixes thereof, with or without 9 μM recombinant human serum albumin (rHSA, Novozymes) were incubated in the presence of a fixed concentration of rhIL-6 (RnD Systems) 2 ng/ml (0.099 nM), TNF 0.4 ng/ml (0.023 nM) or a combination of both cytokines. The pre-mixed samples of inhibitors and cytokines were then transferred to wells containing TF-1 cells. The plates were incubated for 72 h at 37 °C in a humidified 5% CO_2_ atmosphere. During the last four hours of incubation, 10 μl of CCK-8 (Fluka, Sigma Aldrich) was added per well to determine the number of proliferating cells. The absorbance was measured at 450 nm using a microplate reader. The data on cell growth was assessed by nonlinear regression to a three-parameter dose-response curve, and the half maximal inhibitory concentration (IC_50_) was determined using GraphPad Prism 5 (GraphPad Software).

To assess the blocking of the trans-signaling pathway, the following set up was used. HUVECs (Lonza) were grown in EGM-2 bullet kit media (Lonza) and passaged in culture no more than eight times. One day prior to analysis, cells were detached using trypsin/EDTA (Lonza), resuspended in fresh medium and dispensed into 96-well flat-bottomed plates at a density of 2 × 10^4^/cells per well. Cells were then cultured overnight at 37 °C in a humidified 5% CO_2_ atmosphere. In separate plates, serial dilutions of the affibody-ABD fusions and tocilizumab were incubated in the presence of hIL-6 (10 ng/ml; 0.5 nM) and sIL-6Rα (200 ng/ml; 5.6 nM) with or without 9 μM rHSA. The pre-mixed solutions were then transferred to wells containing HUVECs that were incubated for 24 h at 37 °C in a humidified 5% CO_2_ atmosphere. After the incubation period, cell-free supernatant was collected and human monocyte chemoattractant protein-1 (MCP-1) levels determined by sandwich ELISA using the MCP-1 Duoset ELISA development system (RnD Systems) according to the manufacturer's instructions.

### Construction and characterization of AffiMabs

Four different Z*_IL-6_13_*-adalimumab fusions (AffiMabs) were produced by Evitria AG, Switzerland. The anti-IL-6 Z*_IL-6_13_* affibody molecule (denoted AF in **Figure S3**) moiety was genetically fused, via flexible (GGGGS)_3_ linkers, to the N-terminus of either the heavy (HC) or light (LC) chains of adalimumab, resulting in the constructs Z*_IL-6_13_*-HC_Ada_ and Z*_IL-6_13_*-LC_Ada_, respectively, or to the C-termina of the same chains, resulting in the constructs HC_Ada_- Z*_IL-6_13_* and LC_Ada_-Z*_IL-6_13_*, respectively (see **Fig. S3** for more details).

### Affinity determinations

Ten μg/ml solutions of Z*_IL-6_13_*-HC_Ada_, LC_Ada_-Z*_IL-6_13_*, adalimumab and His_6_-Z*_IL-6_13_* were separately prepared in 10 mM NaAc pH 4.5 buffer and used for immobilization of the proteins on a ProteOn GLC chip (Bio-Rad) via amine coupling chemistry. Immobilization levels obtained were ∼1400–2000 resonance units (RU) for the two AffiMabs and adalimumab and 100–200 RU for His_6_-Z*_IL-6_13_*. Repeated three times, a series of 50 nM, 10 nM, 2 nM, 0.4 nM, and 0.08 nM of hIL-6 (RnD Systems) was injected and the responses recorded. In separate triplicate experiments, TNF (RnD Systems) was also injected using the same concentration series. In all cases, the injection was 3 min at a flow rate of 60 μl/min and the dissociation time was 30 min. Buffer response levels obtained for activated/deactivated spots or in-between spots areas were subtracted, as instructed by the manufacturer. The data was analyzed using Bio-Rad Manager Software (BioRad).

### In vivo activity of AffiMab fusion proteins

The Serum Amyloid A (SAA) mouse model was used to explore the in vivo blocking effect of the combined anti-IL-6/anti-TNF Z*_IL-6_13_*-HC_Ada_ AffiMab. The acute phase protein SAA is secreted from liver cells and can be induced by the pro-inflammatory cytokines IL-1, IL-6, and TNF. Due to the sequence homology of the human and mouse cytokines, the human variants are able to act on their corresponding mouse receptors and induce a murine SAA response. The human TNF (hTNF) protein is only able to interact with murine TNF receptor II (TNFRII), and not murine TNFRI. In the experiment, Balb/c mice were injected intraperitoneal (i.p.) with various doses of the Z*_IL-6_13_*-HC_Ada_ AffiMab or the anti-TNF antibody adalimumab (70, 7 or 0.7 mg/kg) 9 h prior to i.p. administration with a mixture of hIL-6 and hTNF each at 2.5 μg/kg (RnD Systems). Sixteen hours post cytokine administration, blood samples were retrieved by orbital puncture and serum was collected. Sera were assessed for the content of murine SAA by ELISA (Tridelta) according to the manufacturer's instructions.
