# Supplementary material for: An affibody-adalimumab hybrid blocks combined IL-6 and TNF-triggered serum amyloid A secretion in vivo
Source: MAbs. 2014 Nov 1;6(6):1598–607. doi: 10.4161/mabs.36089 (PMC4622551; doi:10.4161/mabs.36089)
Supplement: Supplementary_material.pdf [file kmab-06-06-972803-s001.pdf]

SUPPLEMENTARY MATERIAL

to

An Affibody-adalimumab Hybrid Blocks Combined IL-6 and TNF-triggered Serum Amyloid A Secretion *In Vivo*

by

Feifan Yu *et al.*

Supplementary material

		Page
S1	Selection conditions.	3
S2	Initial *in vivo* study of the $Z_{IL\text{-}6\_13}$ affibody in an acute SAA model.	4
S3	Construction and production of AffiMabs.	6
S4	Determination of affinities for two AffiMabs, $Z_{IL\text{-}6\_13}$ or adalimumab to IL-6 and TNF.	9
S5	Comparison of the TNF blocking capabilities of $Z_{IL\text{-}6\_13}$-HC_{Ada} and $Z_{IL\text{-}6\_13}$-LC_{Ada} at a high TNF concentration.	10

S1: Selection conditions

The IL-6 binding affibody proteins were selected from a combinatorial library displayed on bacteriophage (phagemids). During the selection campaign that was performed for four cycles, biotinylated human IL-6 target protein (in one track also murine IL-6 was used for one cycle[(b)]) was used in deceasing concentrations, and the number of washes after capture of phagemid-target complexes on streptavidin beads were successively increased during the selection process. Selection parameters used are summarized in the Table S1 below.

Table S1. Selection conditions.

	**Track 1**	**Track 2**	**Track 3**
**Cycle**	**Target concentration (nM) / No. of washes**	**Target concentration (nM) / No. of washes**	**Target concentration (nM) / No. of washes**
1	100/2	100/2	100/2
2	50/5	10/5	100[(b)]/4
3	25/6	2/8	25
4	10/12	0.5/15	0.5

S2: Initial *in vivo* study of the $Z_{IL\text{-}6\_13}$ affibody in an acute SAA model

An initial study of the anti-arthritic efficacy of the $Z_{IL\text{-}6\_13}$ variant was performed *in vivo* using a mouse model for IL-6 triggered Serum Amyloid A (SAA) protein release. Five groups of Balb/c mice (n=7) were given either none, 0.025, 2.5 or 25 mg/kg body weight of an $Z_{IL\text{-}6\_13}$-ABD fusion protein or 25 mg/kg of a control affibody-ABD fusion protein (Z_{Taq}-ABD) subcutaneously nine hours before an injection of 5 µg/kg body weight of hIL-6. After an additional 22 hours the levels of SAA protein were measured by ELISA (Tridelta) according to the manufacturer's instructions and compared between the different groups. In brief, diluted serum samples were added to SAA-precoated plates together with anti-SAA-HRP. The plates were incubated for 1 hour and then washed four times. TMB substrate was added for 20 min and the reaction was stopped with 2 M H_2SO_4. The absorbance was measured at 450 nm using a microplate reader (Victor3, Perkin Elmer). In animals receiving either nothing of the $Z_{IL\text{-}6\_13}$-ABD fusion or 25 mg/kg of the control affibody-ABD fusion, SAA protein levels increased to levels of approximately 500-600 µg/ml blood compared to controls given only PBS instead of hIL-6 where levels were below 40 µg/ml. In animals given $Z_{IL\text{-}6\_13}$-ABD fusion protein, a clear effect was seen as significantly lower SAA protein levels were measured in a dose-dependent manner (Fig. S2). For the group given the highest dose of $Z_{IL\text{-}6\_13}$-ABD fusion (25 mg/kg body weight), SAA protein levels were as low as for animals given no hIL-6 injection. A control group receiving the highest $Z_{IL\text{-}6\_13}$-ABD fusion (25 mg/kg body weight), but no IL-6, did not show any detectable SAA.

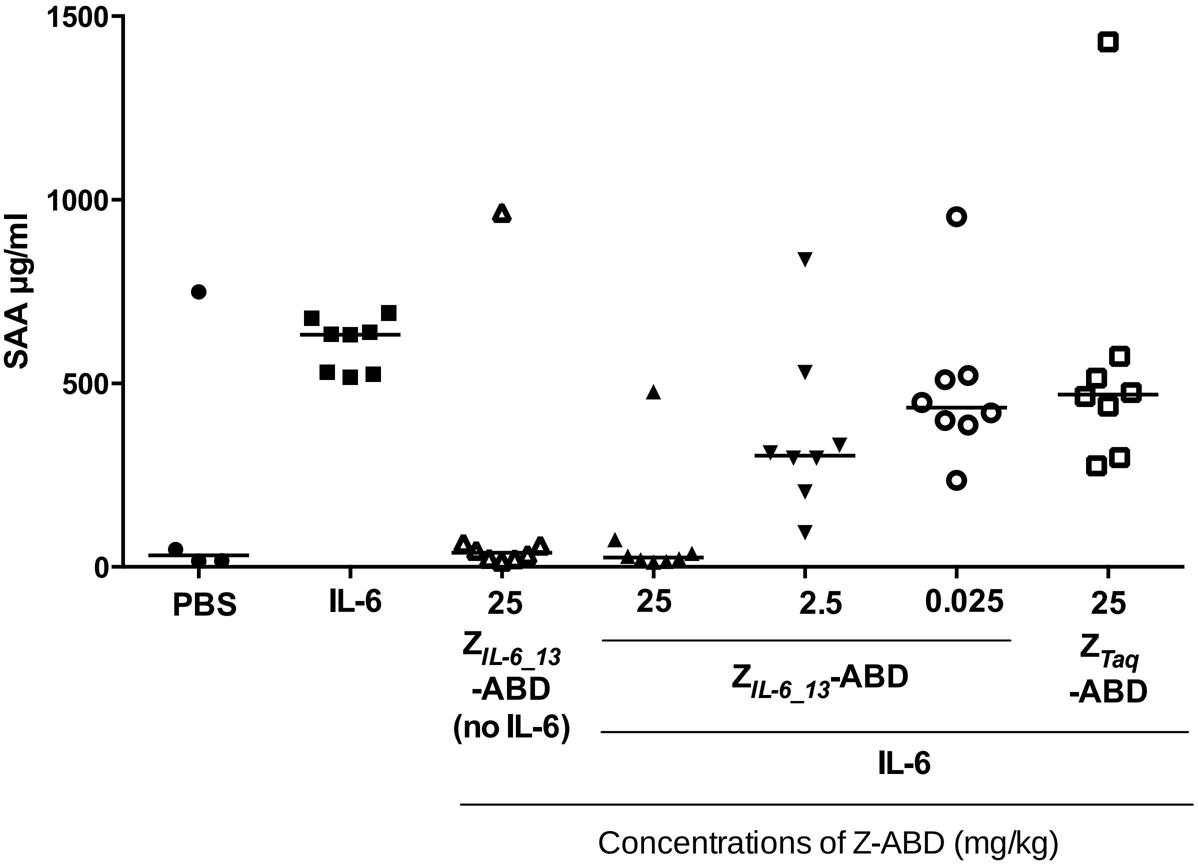

Figure S2. Results from an acute SAA model study in mice for the Z*IL-6_13*-ABD fusion protein. Five groups of Balb/c mice (n=7) were given either 25, 2.5, 0.025 or none mg/kg body weight of an Z*IL-6_13*-ABD fusion protein or 25 mg/kg of a control Z*Taq*-ABD fusion protein subcutaneously nine hours before an injection of 5 µg/kg body weight of hIL-6. After an additional 22 hours the levels of SAA protein were measured by ELISA.

S3: Construction and production of AffiMabs

Four different $Z_{IL\text{-}6\_13}$-adalimumab fusions ("AffiMabs") were designed and produced. The anti-IL-6 $Z_{IL\text{-}6\_13}$ affibody molecule (AF) moiety was genetically fused, via flexible $(GGGGS)_3$ linkers, to both the N-termina of the heavy (HC) and light (LC) chains of adalimumab, resulting in the constructs $Z_{IL\text{-}6\_13}$-HC_{Ada} and $Z_{IL\text{-}6\_13}$-LC_{Ada}, respectively, and to the C-termina of the same chains, resulting in the constructs HC_{Ada}-$Z_{IL\text{-}6\_13}$ and LC_{Ada}-$Z_{IL\text{-}6\_13}$, respectively (Fig. S3A). Production was performed in CHO cells using standard procedures and the AffiMabs purified from the culture supernatants via Protein A-affinity chromatography. The quality and purity of the AffiMabs was analyzed by SDS-PAGE under both non-reduced and reduced conditions (Fig. S3B).

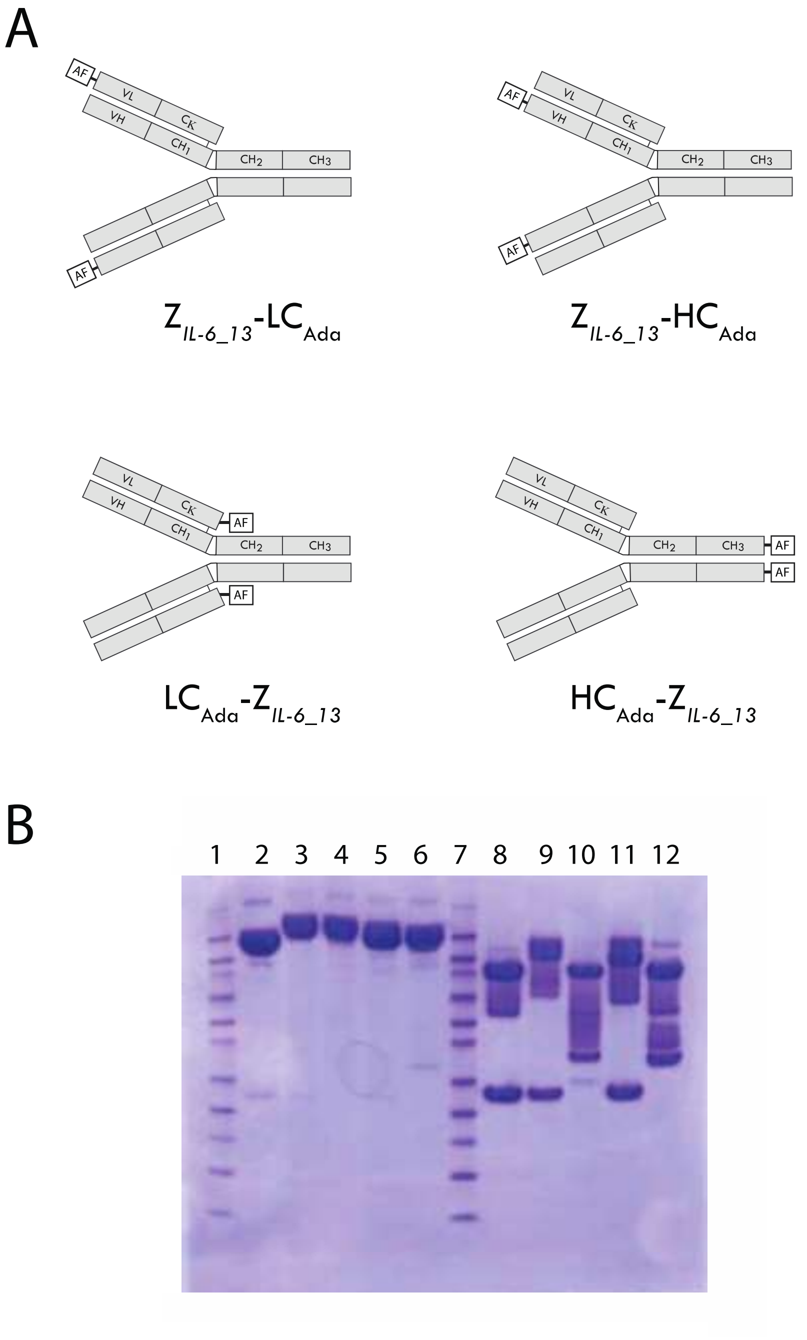

Figure S3. Design and characterization of the four Affimabs. (A): Schematic drawing of the design of the four AffiMab contructs. The designation "AF" depicts the anti-IL-6 affibody variant $Z_{IL\text{-}6\_13}$ sequence. The $Z_{IL\text{-}6\_13}$ affibody sequence was in all constructs genetically linked to anti-TNF mAb adalimumab heavy (HC_{Ada}) or light chains (LC_{Ada}) sequences via a 15 residue $(GGGGS)_3$-linker, positioned either C-terminally (in constructs $Z_{IL\text{-}6\_13}$-LC_{Ada} and $Z_{IL\text{-}6\_13}$-HC_{Ada}) or N-terminally (in constructs LC_{Ada}-$Z_{IL\text{-}6_13}$and HC_{Ada}-

$Z_{IL\text{-}6\_13}$) of the affibody $Z_{IL\text{-}6\_13}$ sequence. (B): SDS-PAGE analysis (12% polyacrylamide gel) of CHO cell produced and protein A affinity purified adalimumab and AffiMab constructs: Lane 1: Marker proteins; lane 2: adalimumab (non-reduced); lane 3: AffiMab $Z_{IL\text{-}6\_13}$-HC_{Ada} (non-reduced); lane 4: AffiMab $Z_{IL\text{-}6\_13}$-LC_{Ada} (non-reduced); lane 5: AffiMab HC_{Ada}-$Z_{IL\text{-}6\_13}$ (non-reduced); lane 6: Affimab LC_{Ada}-$Z_{IL\text{-}6\_13}$ (non-reduced); lane 7: Marker proteins; lane 8: adalimumab (reduced); lane 9: AffiMab $Z_{IL\text{-}6\_13}$-HC_{Ada} (reduced); lane 10: AffiMab $Z_{IL\text{-}6\_13}$-LC_{Ada} (reduced); lane 11: AffiMab HC_{Ada}-$Z_{IL\text{-}6\_13}$ (reduced) and lane 12: AffiMab LC_{Ada}-$Z_{IL\text{-}6\_13}$ (reduced).

S4: Determination of affinities for two AffiMabs, $Z_{IL\text{-}6\_13}$ or adalimumab to IL-6 and TNF.

The affinities for IL-6 and TNF were determined using proteins and conditions as described in the main text (see Materials and Methods section). In the Table S4 below are summarized the measured association rate constants, dissociation rate constants and calculated dissociation constants interactions between the indicated constructs and IL-6 and TNF, respectively.

Table S4. Summary of affinity determinations. Summary of measured association rate constants, dissociation rate constants and calculated dissociation constants interactions between the indicated constructs and IL-6 and TNF, respectively.

IL-6

	k_a (M^{-1} s^{-1})	k_d (s^{-1})	K_D (M)
$Z_{IL\text{-}6\_13}$-HC_{Ada}	$3.16 \pm 0.02 \times 10^5$	$2.28 \pm 0.30 \times 10^{-4}$	$7.19 \pm 0.80 \times 10^{-10}$
LC_{Ada}-$Z_{IL\text{-}6\_13}$	$1.40 \pm 0.03 \times 10^5$	$1.30 \pm 0.20 \times 10^{-4}$	$9.25 \pm 1.20 \times 10^{-10}$
His_6- $Z_{IL\text{-}6\_13}$	$3.07 \pm 0.05 \times 10^5$	$1.53 \pm 0.10 \times 10^{-4}$	$4.97 \pm 0.30 \times 10^{-10}$

TNF

	k_a (M^{-1} s^{-1})	k_d (s^{-1})	K_D (M)
$Z_{IL\text{-}6\_13}$-HC_{Ada}	$6.21 \pm 0.04 \times 10^5$	$1.05 \pm 0.00 \times 10^{-4}$	$1.70 \pm 0.02 \times 10^{-10}$
LC_{Ada}-$Z_{IL\text{-}6\_13}$	$8.42 \pm 0.05 \times 10^5$	$1.10 \pm 0.02 \times 10^{-4}$	$1.31 \pm 0.03 \times 10^{-10}$
adalimumab	$7.41 \pm 0.06 \times 10^5$	$1.60 \pm 0.10 \times 10^{-4}$	$2.16 \pm 0.01 \times 10^{-10}$

S5: Comparison of the TNF blocking capabilities of $Z_{IL\text{-}6\_13}$-HC_{Ada} and $Z_{IL\text{-}6\_13}$-LC_{Ada} at a high TNF concentration.

The two AffiMab constructs $Z_{hIL\text{-}6\_13}$-HC_{Ada} and $Z_{hIL\text{-}6\_13}$-LC_{Ada} were compared to adalimumab in a TF-1 cell assay for their capacities to block the effect of the relatively high TNF concentration of 71.5 pM. Cells were cultured in RPMI1640 with L-glut (Lonza) supplemented with 10% FCS (Gibco), Pen-Strep (Lonza) and 2 ng/ml rhGM-CSF (RnD Systems). Prior to analysis, cells were washed twice in RPMI1640 in the absence of rhGM-CSF. Cells were dispensed into 96 well flat bottomed plates at a density of 4×10^4/cells per well. In separate plates serial dilutions of the AffiMab molecules or adalimumab were incubated in the presence of a fixed concentration of TNF 1.2 ng/ml (0.072 nM). The pre-mixed samples of AffiMab molecules or adalimumab and TNF were then transferred to wells containing TF-1 cells. The plates were incubated for 72 hours at 37°C in a humidified 5% CO_2 atmosphere. During the last four hours of incubation 10 µl of CCK-8 (Fluka, Sigma Aldrich) was added per well to determine the number of proliferating cells. The absorbance was measured at 450 nm using a microplate reader. The experiment showed that both AffiMabs were capable of effective blocking of the growth stimulating TNF signal, but that the $Z_{IL\text{-}6\_13}$-HC_{Ada} construct was more efficient than the $Z_{IL\text{-}6\_13}$-LC_{Ada} construct with an effect in parity with adalimumab included as reference (Fig. S5).

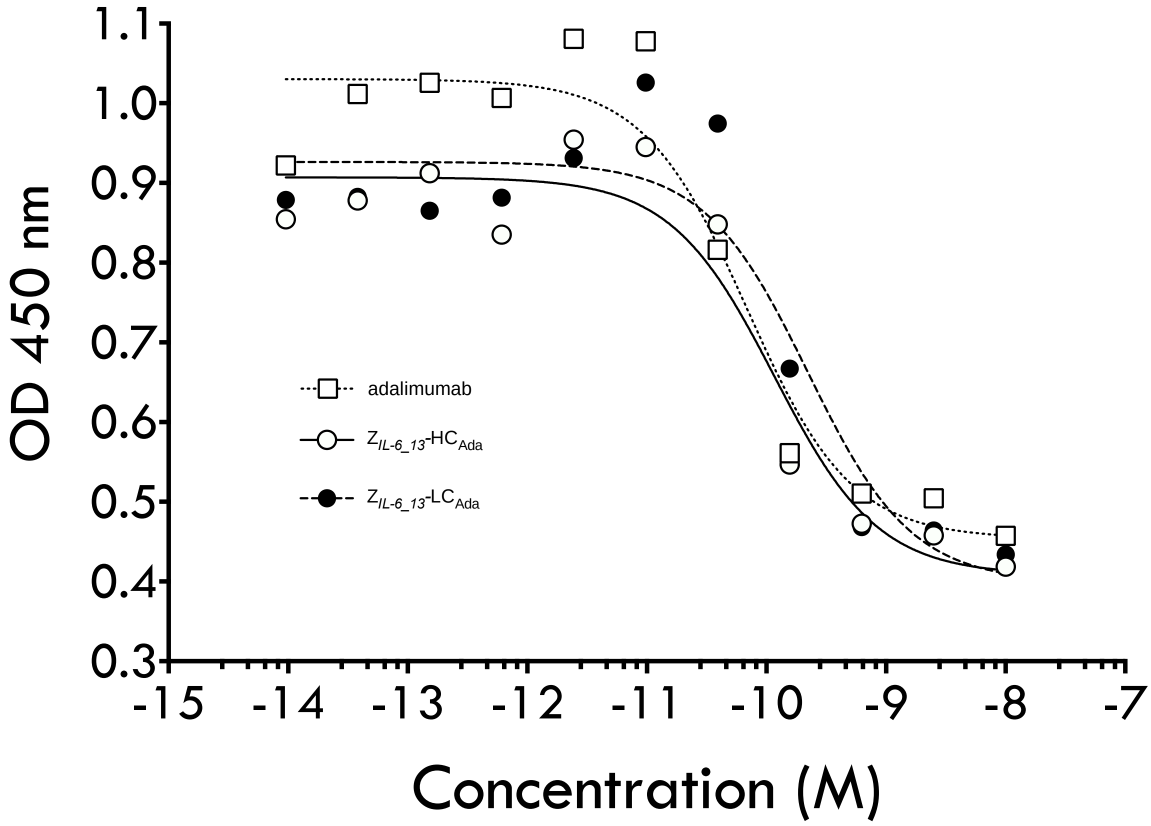

Figure S5. Comparison of Z$_{IL\text{-}6_13}$-HC$_{\text{Ada}}$, Z$_{IL\text{-}6_13}$-LC$_{\text{Ada}}$ AffiMabs and adalimumab for blocking of a high TNF concentration. Results from an analysis of the capability of Z$_{IL\text{-}6_13}$-HC$_{\text{Ada}}$, Z$_{IL\text{-}6_13}$-LC$_{\text{Ada}}$ AffiMabs or adalimumab to inhibit the growth of TNF stimulated TF-1 cells. The TNF concentration was 71.5 pM.
